# Nutrition, aging and cancer: lessons from dietary intervention studies

**DOI:** 10.1186/s12979-016-0069-9

**Published:** 2016-04-07

**Authors:** Giuseppe Carruba, Letizia Cocciadiferro, Antonietta Di Cristina, Orazia M. Granata, Cecilia Dolcemascolo, Ildegarda Campisi, Maurizio Zarcone, Maria Cinquegrani, Adele Traina

**Affiliations:** Division of Research and Internationalization, ARNAS-Civico Di Cristina e Benfratelli, Palermo, Italy; Research Laboratories Dr. Nicola Locorotondo, Palermo, Italy; Clinical Pathology, “G. DI Cristina” Pediatric Hospital ARNAS-Civico Di Cristina e Benfratelli, Palermo, Italy; The Diana Project, National Cancer Institute, Milan, Italy

## Abstract

There is convincing epidemiological and clinical evidence that, independent of aging, lifestyle and, notably, nutrition are associated with development or progression of major human cancers, including breast, prostate, colorectal tumors, and an increasingly large collection of diet-related cancers. Mechanisms underlying this association are mostly related to the distinct epigenetic effects of different dietary patterns. In this context, Mediterranean diet has been reported to significantly reduce mortality rates for various chronic illnesses, including cardiovascular diseases, neurodegenerative diseases and cancer. Although many observational studies have supported this evidence, dietary intervention studies using a Mediterranean dietary pattern or its selected food components are still limited and affected by a rather large variability in characteristics of study subjects, type and length of intervention, selected end-points and statistical analysis. Here we review data of two of our intervention studies, the MeDiet study and the DiMeSa project, aimed at assessing the effects of traditional Mediterranean diet and/or its component(s) on a large panel of both plasma and urine biomarkers. Both published and unpublished results are presented and discussed.

## Background

Cancer represents today the second leading cause of death worldwide after cardiovascular diseases, despite the fact that cancer mortality rates have been declining since 90ies because of either early diagnosis/screening programs or an increasingly larger array of therapeutic options [1].

In this context, the continuous rise in life expectancy has significantly contributed to the steady increase of cancer risk among general population [2]. However, mechanisms underlying both aging and cancer processes are still subjects of controversies more than consensuses, though cancer and aging seem to share common, rather than antagonistic, etiologies [3]. Notwithstanding, it ought to be emphasized that the onset of many chronic illnesses, including cancer, is occurring today at an average age earlier than ever, with a progressive leftward shift in the age of onset of various diseases including diabetes, cancer and obesity [4, 5]. This alarming figure would implies that, at least as far as cancer is concerned, the increasing incidence of human malignancies is not only related to the current increase in life expectancy, but other key risk factors, mostly related to lifestyle and environment, must be taken into account.

A number of both epidemiological and clinical studies strongly support the association between nutrition and development or progression of major human cancers, including breast, prostate, and colorectal tumors, but also many other tumor types have been recently included in a hypothetical list of diet-related cancers (reviewed in [6]).

There are many dietary components that have been implicated as either protective or promoting factors in cancer development. Several bioactive food components, including polyphenols, selenium, methyl-group donors, retinoids, mono- and poly-unsaturated fatty acids, isothiocyanates and allyl compounds, have all been claimed to have cancer prevention potential [7]. Although these compounds may impact upon a variety of different cellular processes, such as DNA repair, growth and differentiation, programmed cell death, oxidative stress, inflammation and so forth, in recent years the epigenome has been implicated as the primary target for nutrition-induced changes of gene expression and function [8].

By definition, epigenetics comprises heritable changes in gene expression with no alteration of DNA sequence [9]. Major epigenetic mechanisms include DNA methylation, histone modification and RNA interference (RNAi) [10]. There is mounting evidence that all these epigenetic processes can be implicated and act in synergy with genetic alterations during carcinogenesis and tumor progression [11, 12]. However, contrary to genetic mutations, epigenetic abnormalities are potentially reversible and can be targeted by both cancer prevention and therapeutic strategies [13].

Convincing evidence is accumulating that many bioactive dietary components may extensively modulate epigenetic mechanisms, eventually leading to a rapid and effective regulation of gene expression and function in response to nutritional changes. In this respect, the term epigenetic diet has been introduced to indicate the consumption of foods, such as soy, grapes, cruciferous vegetables and green tea, that affect epigenetic mechanisms to protect against cancer and aging [14].

It is noteworthy that unbalanced maternal nutrient intake may severely impact on fetal epigenome early during in utero development. There is increasing evidence that nutritionally-induced epigenetic alteration of the offspring’s epigenome may be responsible for higher susceptibility to cancer development later in life [15] and that several epigenetic marks can be inherited and reshape developmental and cellular features over generations, a phenomenon referred to as epigenetic inheritance [16].

## Mediterranean diet & disease risk: observational and intervention studies

The term Mediterranean Diet was originally coined after Angel Keys and his colleagues ascribed the significantly lower rates of coronary heart disease observed in Mediterranean countries, including Italy and Greece, as compared to “northern” countries, such as Netherlands, Finland and USA, to various factors and, most notably, to the protective role of what they later called the “good Mediterranean diet” and its typical food components [17, 18].

Although there are various definitions of the Mediterranean Diet (MD) in literature, it is difficult to describe in details a Mediterranean dietary pattern and its components. According to Simopoulos [19], the term “Mediterranean Diet” is in fact a misnomer, simply because there are many different “Mediterranean diets”, based on cultural, ethnical, religious and economical diversities among different populations and countries belonging to the Mediterranean basin. However, the distinct Mediterranean Diets generally share some major features, precisely: (a) a high consumption of whole grains (over 60 % of total caloric intake), (b) a high consumption of vegetables, fruits, and legumes; (c) extravirgin olive oil for over 70 % of dietary fat; (d) a regular consumption of fresh fish (especially marine blue species); (e) a low intake of saturated animal fats, red and processed meat, poultry, dairy products; and (f) with the exception of muslim populations, a regular but moderate consume of red wine during main meals.

Adherence to a Mediterranean dietary pattern has generally been associated with a decreased risk of dying for most non-communicable diseases, including cardiovascular diseases, cancer, neurodegenerative diseases. Despite the difficulty to assess the extent of adherence to this dietary pattern and the limited number of studies exploring in depth the impact of Mediterranean Diet on chronic diseases and relevant pathogenetic mechanisms, both meta-analyses and systematic reviews of the existing literature have highlighted that Mediterranean Diet has a significant protective role on risk of cardiovascular and neurodegenerative disease, diabetes and cancer [20–22].

As far as dietary interventions are concerned, the protective role of Mediterranean Diet against chronic diseases has been challenged in a variety of studies.

The Lyon Diet Heart Study, a randomized secondary prevention trial aimed at testing whether a Mediterranean diet may reduce the rate of recurrence after a first myocardial infarction, indicated that the adherence to a Mediterranean dietary pattern has a significant protective role against secondary cardiac events, including death and nonfatal myocardial infarction, and that this effect is maintained up to 4 years after the first infarction, independent of major traditional risk factors, such as high blood cholesterol and blood pressure [23].

After such a pioneering observation, a number of dietary intervention studies have investigated the potential role of Mediterranean diet in the prevention of cancer, obesity, cardiovascular and neurodegenerative diseases. Serra-Majem and Estruch [24], in a systematic review of 35 experimental studies, indicated that Mediterranean diet has favorable effects on lipoprotein levels, endothelium vasodilatation, insulin resistance, metabolic syndrome, antioxidant capacity, myocardial and cardiovascular mortality, and cancer incidence in obese patients and in those with previous myocardial infarction.

In a recent systematic review of 11 randomized clinical trials for the Cochrane Collaboration, Rees and colleagues [25] concluded that, despite the limited evidence available, Mediterranean diet positively affects cardiovascular risk factors, including total cholesterol and low-density lipoprotein (LDL) cholesterol. More recently, Sleiman and associates [22] systematically reviewed 24 studies (including cross-sectional, prospective and controlled clinical trials) to show that Mediterranean diet shows favorable effects on both glycemic control and cardiovascular disease, while some degree of controversy remains on other issues, such as obesity.

In the Moli-sani study, a large prospective cohort stud, a Mediterranean-like diet was significantly associated with lower values of glucose, lipids, CRP, blood pressure and 10-year cardiovascular risk, while the consumption of healthy foods with high content in antioxidant vitamins and phytochemicals correlated with lower blood pressure and CRP plasma levels [26].

The PREDIMED study, a multicenter, randomized, primary prevention trial, showed that Mediterranean Diet, supplemented with either extravirgin olive oil or nuts, has favorable effect on blood pressure, insulin sensitivity, lipid profiles, lipoprotein particles, inflammation, oxidative stress, and carotid atherosclerosis [27].

As reviewed by Ostan and colleagues [28], there is consisting evidence that nutrition modulates multiple interconnected processes that play a major role in both carcinogenesis and inflammatory responses, including free radical production, NF-κB activation, expression of inflammatory cytokines, and the eicosanoids pathway [29]. In particular, Mediterranean Diet may positively impact on the so called “inflammaging” through either epigenetic mechanisms (that include chromatin remodeling, DNA methylation and miRNAs) or preservation of gut microbiota homeostasis.

## Primary prevention of breast cancer: The MeDiet study

Breast cancer is the commonest malignancy in women and the second most common neoplasm worldwide, accounting for over 25 % of all female cancers [30]. It is worth noting that breast cancer incidence and mortality rates vary greatly across several countries, respectively ranging from 25.6 to 95.3/100.000 and from 5.2 to 19.4/100.000. This large variations may be ascribed to differences in both lifestyle and, notably, dietary habits among different geographical areas. Results from migrant studies have indicated that breast cancer incidence dramatically changes in Asians migrating to USA and in their offspring, with a steady increase from first to second and third generation [31], suggesting that international variations in breast cancer incidence represent a consequence of differences in lifestyle or environmental factors rather than genetic differences.

Estrogens are primarily implicated in both development and progression of human breast cancer, as either genotoxic or promoting agents [32–34]. However, there is little information on the impact that lifestyle, especially nutrition, produces on estrogen levels and metabolism and, hence on breast cancer risk.

Some years ago, we conducted the MeDiet study, a randomized, dietary intervention trial aiming to determine the effects of a traditional Mediterranean diet on endogenous estrogens in healthy postmenopausal women [35].

Out of 230 healthy female volunteers, 115 women were found to be eligible and were enrolled in the study based on serum testosterone levels equal or greater than 0.14 mg/ml (median value), an arbitrary cut-off value that has previously been reported to identify women at higher risk of developing breast cancer [36, 37]. After recruitment, study subjects were randomized into a dietary intervention (*n* = 58) and a control (*n* = 57) group. Women in the intervention group adopted a traditional, controlled Mediterranean diet for 6 months, while women in the control group continued to follow their regular diet. The intervention group women were instructed, through a weekly cooking course, to use and cook food components of the traditional Sicilian (Mediterranean) diet, including: whole cereals, legumes, seeds, blue fish, extravirgin olive oil, vegetables, and other Mediterranean seasonal food. Conversely, these subjects were advocated to reduce and/or avoid use of refined carbohydrates and additional animal fat, and to limit the use of salt.

Before (*baseline*) and after dietary intervention, women in both control and intervention groups undertook the following: (a) compiled a food frequency questionnaire originally developed for the EPIC study [38]; (b) collected both fasting blood samples and 12 h urine samples; (c) measured anthropometric indexes, including height, weight, waist-to-hip ratio.

Urine samples were of special interest since our early studies have indicated that metabolic profiles of urinary estrogens could be used to discriminate breast cancer patients in relation to their estrogen status, response to hormone treatment and prognosis [39]. Furthermore, urinary profiles of estrogens appear to be representative of intratissue estrogen content, as defined in our previous studies [40].

Profiles of endogenous estrogens in urine of both intervention and control women were assessed using a high performance liquid chromatography (HPLC) system, with both a photodiode array and an on line electrochemical detection.

At baseline, no significant difference was observed in urinary levels of individual estrogens comparing intervention and control women. It is important to note that the majority of urinary estrogens was represented by hydroxy and methoxy derivatives of both estradiol (E2) and estriol (E3), rather than by parent estrogens (E2, E3, and estrone – E1), in both control (75 %) and intervention (84 %) groups. This finding is in accordance with results of our early studies indicating that a high proportion of estrogens in urines of breast cancer patients is represented by hydroxylated metabolites of circulating (mainly E2 and E1) estrogens, referred to as “minor” or “unusual” metabolites [39]. In addition, this picture is cognate to what we have observed when measuring intratissue profiles of estrogens in both normal and malignant human mammary gland, where hydroxy and/or methoxy derivatives of either E2 or E1 represent the vast majority of tissue estrogens while parent hormones (E2, E1 and E3) account for a mere 5.2 % and 4.3 % in *nontumoral* and malignant breast, respectively [40]. More importantly, some of these compounds, notably hydroxylated metabolites and semiquinone or quinone derivatives of either E2 or E1 have been implicated as cancer-initiating or –promoting agents in breast carcinogenesis and tumor progression [32–34].

**Fig. 1:**
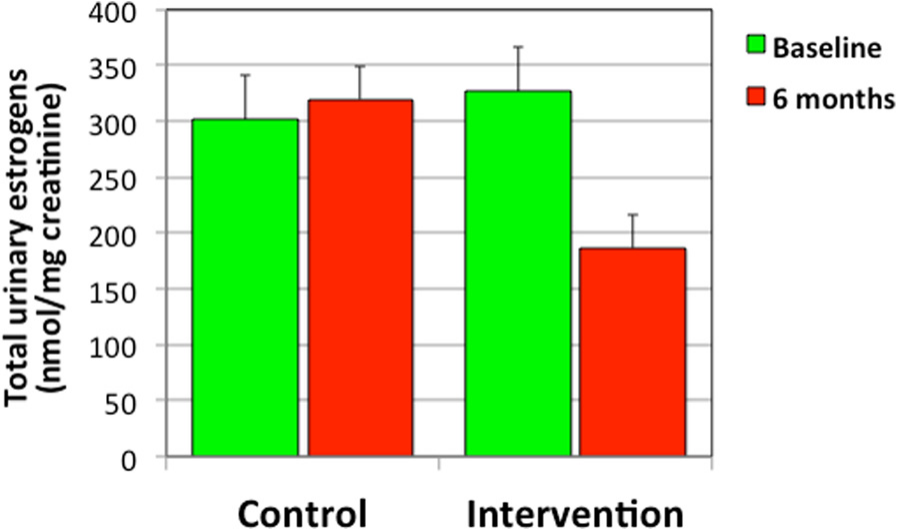
Levels of total urinary estrogens in both control and intervention women at baseline and after 6 months in the MeDiet study. Average values ± SDs are represented

**Fig. 2:**
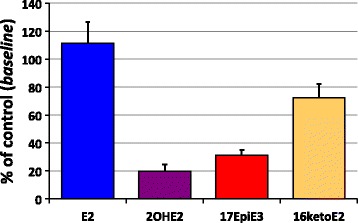
Changes of estrogen concentrations in intervention women after 6 months in the MeDiet study. Data represent % of control, as compared with baseline values, ±SDs of estradiol (E2), 2hydroxy-estradiol (2OHE2), 17epi-estriol (17epiE3) and 16keto-estradiol (16ketoE2)

After 6 months, as expected, control women did not show any major change, whilst women in the intervention group showed a significant reduction (over 40 %, *P* < 0.02) of total estrogen levels (Fig. 1). Interestingly, this modification was in consequence of a remarkable reduction of estrogen metabolites, including hydroxy- and keto-derivatives of E2 or E3, notably 2hydroxy-E2 (2OHE2), 17EpiE3 and 16KetoE2, whose concentrations decreased by 80, 70 and 27 %, respectively (Fig. 2). At baseline, these three compounds represented the majority of urinary estrogens in both control (57 %) and intervention (68 %) women; after 6 months their levels dropped drastically in the intervention group (54 %), while remained unchanged in the control group (91 %). Conversely, E2, which represented a mere 0.2 % of total urinary estrogens, showed a limited though statistically significant increase in the intervention group (see Fig. 2), presumably because of its decreased biotransformation into 2OHE2 and 16KetoE2 that occurs in the intervention group.

**Fig. 3:**
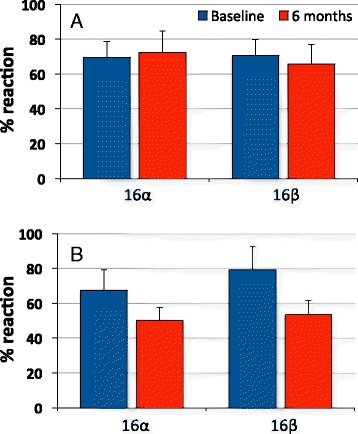
Extent of 16α and 16β hydroxylation in control (**a**) and intervention (**b**) women of the MeDiet study. Data represent average ± SD percent values of individual reaction at both baseline and after 6 months

Overall, this MeDiet study has provided convincing experimental evidence of two-fold value. In the first place, it has further highlighted the fact that both hydroxy and methoxy estrogen derivatives account for the vast majority of endogenous estrogens in human urine and that urinary estrogen profiles can be considered much closer to intratissue estrogen content than respective plasma values, where only parent estrogens (E2, E1, E3), that represent a limited fraction (5–8 %) of total endogenous estrogens, could be measured. Secondly, this study has clearly indicated that traditional Mediterranean diet may reduce the risk of developing breast cancer also through its effects on estrogen metabolism, whereby hydroxylation of E2 at C2, C16α and C16β position, a process eventually leading to the formation of genotoxic metabolites, is remarkably decreased in the intervention group but remains unchanged in the control group (Fig. 3).

## Primary prevention of cancer and *noncommunicable* diseases: the DiMeSa project

Today the crisis of the agrifood sector across several geographical European areas, including our own, combined with the recent economic crisis running at regional, national and community level, is featured by extremely critical aspects, mainly residing in the limited innovation potential of companies and enterprises, the lack of integration with public-private research institutions, the insufficient systematization and organization of the existing resources in an extended territorial networking. This criticism results into increasing difficulties of small/medium enterprises (SMEs) to be present in both domestic and foreign markets with characteristics of quality and competitiveness.

On the other hand, several epidemiological studies clearly indicate that all Western regions, including our country, are facing a dramatic phenomenon consisting of real epidemics of chronic diseases (cardiovascular, cerebro-vascular, and respiratory diseases, diabetes, obesity, metabolic syndrome) and tumors, whose causes are largely attributable to (removable) lifestyle risk factors, notably diet [41]. In particular, the gradual abandonment of the traditional principles and food of Mediterranean diet, that have protected for decades populations of Southern Europe, including Italy, Greece and Spain, is leading to a significant increase in incidence rates of these diseases in our region, where today, paradoxically, the highest European percentages of obese and/or overweight adolescents are being observed [42]. Since mortality from these diseases, that we might rightfully define lifestyle-based, is steadily declining since 90ies, thanks to more effective early diagnosis and to advancements in treatment options, we are witnessing an alarming “scissor” phenomenon, where a progressive increase in the number of new cases/year is accompanied by a significant reduction number of deaths, with a remarkable and continuous increase in the number of chronically ill individuals (prevalence) [41]. This phenomenon, has an economic, social and health impact of enormous importance worldwide, to the point that the World Health Organization (WHO), along with all major health institutions, have proposed primary prevention as the only effective approach to halt and eventually reverse it and launched a pluriannual (2013–2020) action plan for the prevention and control of non-communicable diseases based on a comprehensive intersectorial strategy targeting the removal of primary risk factors for these diseases, notably western diet [43]. Based on this combined consideration, promoting both production and competitiveness of traditional food products in regional, domestic and foreign markets through a series of activities aimed at increasing their health and/or nutraceutical potential, to clinically validate their effects on both health and chronic disease(s), and to enable rapid technological transfer and industrial development of either processes or products would represent a systemic strategy of high impact in the short, medium and long term for the important expected outcome from an economic, technological and healthcare standpoint.

Armed with this conception, in October 2012 we embarked on a large regional project, funded by the Italian Ministry of University and Research (MIUR), leaded by the The AgroBioPesca Technology Cluster, called “DiMeSa - Valorization of typical products of Mediterranean Diet and their use for health and nutraceutical purposes”, where DiMeSa stands for **Di**eta **ME**diterranea e **Sa**lute, simply Mediterranean Diet and Health.

This project was mainly aimed at multiplying the attractiveness and market capacities of the traditional products of Mediterranean diet in regional agriculture through the development and implementation of industrial research and experimental development activities that would eventually lead to improve their health potential and that, at the same time, would validate scientifically the relationship existing between selected Mediterranean food products and health, both in terms of maintaining a well-being state and, especially, of primary disease prevention.

The DiMeSa project is arranged into four major objectives, precisely:

1. the analysis and identification of traditional food processes and the development of innovative biotechnological protocols for the production of food with high nutritional and health potential, including extra virgin olive oil, cereals or vegetables and their derivatives;
2. the definition and implementation of procedures and methodological approaches for the production of functional foods (exra virgin olive oil, pasta, juices) through their enrichment (*functionalization*) with natural substances and/or plant/byproducts extracts having high health potential and their distribution through innovative vending machines;
3. the clinical validation of specific health claims through the conduction of randomized, controlled clinical trials to assess the health effects of selected *functional* food products on cohorts of either healthy, high-risk or diseased study-subjects through the evaluation of the impact of dietary intervention on some clinical and biomolecular end-points, such as: a) anthropometric measures; b) immunological markers of inflammation; c) oxidative stress and endothelial function; d) hormonal profiles and gene expression;
4. the economic evaluation of the concept, traceability and industrial scale-up of either prototypal products or processes aiming to allow their immediate industrialization and successful marketing.

The project itself is a multicenter study, characterized by a rather large partnership, that included regional Universities and other public research institutions, on one end, and small/medium enterprises (SMEs), on the other (see Table 1). The project run for 39 months and ended just recently, by December 2015.

**Table 1:** The DiMeSa Project: partnerships

Public	SMEs
• University of Palermo - SAF - DIFI - DIBIMEF - DIMIS - STEMBIO - CGA - DICGIM • University of Catania - Dip. Scienze del Farmaco - D3Di • University of Messina - Dip. Clinico-Sperimentale di Medicina e Farm. • National Research Council (CNR) - IBF-Palermo - IBIM-Palermo - ISAFOM-UOS-Catania • Ballatore Consortium • CoRiSSIA • IZSS	• CoRiSvI - Oleificio San Calogero - Az. Agr. Angela Consiglio - Azienda “GeOlive” Belice - Pastificio Tomasello SpA - Laboratorio di Ricerche Locorotondo • Innova Agro Sicilia - Molino di Sicilia SrL - Agriplast SrL - Medivis • Agroindustry Advanced Technologies (AAT)

**Fig. 4:**
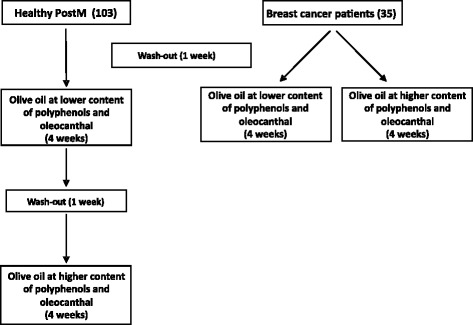
Flow chart of a randomized clinical trial in the DiMeSa project to assess the effects of extravirgin olive oil on selected parameters in both healthy postmenopausal women and breast cancer patients. For explanation see text

In this framework, we have conducted a randomized clinical trial for the assessment of the health impact of extravirgin olive oil (EVO) on 2 cohorts of study subjects represented by healthy postmenopausal women and patients with breast cancer. All study subjects were recruited at the Azienda di Rilievo Nazionale e di Alta Specializzazione (ARNAS) - Civico, Di Cristina, Benfratelli. Overall, 103 healthy postmenoausal women and 35 breast cancer patients were enrolled in the study. Two different mono-cultivar EVOs, one at lower (BL) and one at higher (CS) content of polyphenols and oleocantal, were used in the study; both EVOs were produced by the SAF (Scienze Agrarie e Forestali) Department of Palermo University, under the supervision of Prof. Tiziano Caruso. As regards healthy women, after an initial 1 week wash-out period (“no EVO” week), the subjects consumed a daily amount of 30 ml of the BL EVO for 4 weeks, followed by another 1 week wash-out period (“no EVO”) and an additional 4 weeks intervention with the CS EVO, as illustrated in Fig. 4. Conversely, breast cancer patients were randomized into one BL EVO and one CS EVO intervention group that consumed daily amounts of 30 ml of either BL or CS EVO for 4 weeks (see Fig. 4). Both healthy and breast cancer study subjects, before and after any EVO intervention, undertook the following: (a) compiled a food frequency questionnaire originally developed for the EPIC study [38]; (b) measured anthropometric indexes, including height, weight, waist-to-hip ratio; (c) were administered psychometric tests (HADS, SF-36); (d) collected both fasting blood samples and 12 h urine samples. These latter were collected to determine the potential effect of dietary intervention on an array of both plasmatic and serum biomarkers, the expression profiles of a set of previously selected genes, the whole miRNome and the urinary profile of sex steroid hormones.

Biological samples (plasma, serum, urine) were collected and stored in a biobank that, as far as the EVO clinical trial is concerned, included 5364 plasma/serum aliquots and 756 urine aliquots. A web-based study database was also created and data processed using advanced statistical analysis.

Currently, only preliminary results of the trial are available, concerning specifically a series of plasmatic biomarkers, before and after dietary intervention, in both healthy women and breast cancer patients. As reported in Table 2, consumption of BL EVO resulted in significant changes of various plasmatic biomarkers in both healthy subjects and breast cancer patients. In particular, the reduction of glycemia, insulinemia and total cholesterol levels appears of special interest. On the other hand, the consumption of CS EVO produced several modifications in selected biomarkers (see Table 3), including a significant increase of HDL cholesterol and reduction of LDL cholesterol. Interestingly, this EVO also induced a marked decrease of plasmatic levels of estradiol. It appears noteworthy that, when comparing the two EVOs, BL EVO appeared to be more effective in reducing glycemia, while CS EVO proved to be more effective in decreasing plasmatic estradiol (see Table 4). This would imply that different EVOs may have a distinct impact on either glycemic control or hormonal (sex steroid) status, also depending on the cultivar and on the phenology of fruit ripening (the ealier the stage, the greater the content of polyphenols).

**Table 2:** Effects of BL EVO on plasmatic biomarkers in both healthy postmenopausal women and breast cancer patients

Variable	Baseline	After	*p*-value^a^
Azotemia	30.85	28.48	0.002
Uricemia	4.17	4.29	0.001
Glycemia	85.35	83.59	0.021
Insulinemia	10.33	8.79	<0.001
Total cholesterol	207.48	197.12	<0.001
Gamma GT	21.90	24.50	0.001
Total Proteinemia	7.00	6.91	0.005
Sideremia	76.95	67.02	<0.001

^a^paired *T* test, ANOVA

**Table 3:** Effects of CS EVO on plasmatic biomarkers in both healthy postmenopausal women and breast cancer patients

Variable	Baseline	After	*p*-value^a^
Cretininemia	0.69	0.63	<0.001
Uricemia	4.23	4.43	0.002
Glycemia	89.16	88.48	0.023
Glycated hemoglobin	5.64	5.51	<0.001
HDL cholesterol	57.87	59.31	0.023
LDL cholesterol	119.62	102.12	0.047
Testosterone	0.39	0.36	0.033
Estradiol	31.40	23.95	0.002

^a^paired T test, ANOVA

**Table 4:** Comparison of the effects of BL and CS EVO on glycemia and estradiol levels in both healthy postmenopausal women and breast cancer patients

Variable	BL EVO	CS EVO	*p*-value^a^
Glycemia	83.59	88.48	0.023
Estradiol	37.21	23.95	0.027

Results of gene expression, microRNA profiling and patterns of urinary sex steroids are currently under statistical analysis and are awaited with great expectation and interest.

## Conclusions and perspectives

**Fig. 5:**
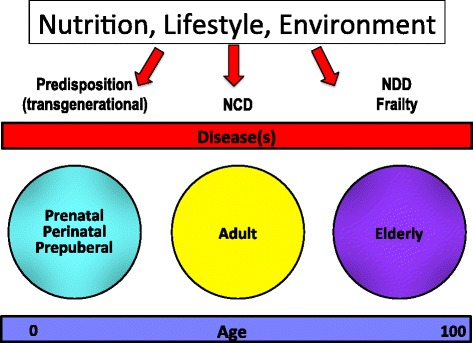
A schematic diagram representing the impact of nutrition, lifestyle and environment on health and diseases throughout life cycle. In particular, the potential effects during pre- and peri-natal life, adulthood and aging are depicted (see text). NCD, noncommunicable diseases; NDD, neurodegenerative diseases

Doubtlessly, environment and lifestyle play a major role in human health and aging. An amazing array of environmental factors and nutrition may have in fact a major impact upon cellular and molecular processes, challenging severely cell adaptation capacities and the maintenance of tissue homeostasis. In this scenario, various aspects related to diet may be of crucial importance, including caloric intake, meal timing, balance of macro- and micro-nutrients, microbiome regulation [44]. Recently, WHO Europe launched a campaign for health promotion through a life-course approach, with the motto “Act Early, Act on Time, Act Together”. This approach encompasses virtually all key health issues and relevant determinants throughout the entire life cycle, including pre- and perinatal life, adulthood and aging, as depicted in Fig. 5.

Data from our dietary intervention studies have underlined some interesting aspects related to both mechanisms underpinning biological and clinical effects of nutrition, on one hand, and specific activities of Mediterranean food components, on the other. In particular, the MeDiet study has provided evidence that Mediterranean diet may regulate estrogen metabolism in postmenopausal women in a way that the formation of potentially harmful genotoxic compounds is remarkably reduced, while levels of parent hormone estradiol are slightly increased; this would imply that traditional Mediterranean food reduces the risk of developing breast cancer while limiting the side-effects of estrogen withdrawal in menopause.

On the other hand, the DiMeSa project has indicated that technological innovation and prototypical industrialization of either processes or products could be used to obtain traditional Mediterranean food having high health potential and market capacities. Precisely, the production of monocultivar extravirgin olive oils (EVOs) has revealed that selected EVO(s) may have a differential activity on different cellular and metabolic processes, eventually leading to produce highly characterized EVOs with a preferential use for the prevention and care of distinct chronic diseases.

Nevertheless, further large, multicentric and long-term, prospective intervention studies are yet necessary to dissect the real effects of a strictly defined Mediterranean dietary pattern on both individual risk of noncommunicable diseases and the underpinning action mechanisms.
